# Tobacco use related attitudes and behaviors in Indian Adolescents: association with school-based prevention education

**DOI:** 10.15171/hpp.2017.24

**Published:** 2017-06-14

**Authors:** Jagdish Khubchandani, Manoj Sharma, David Huston, Jay Tahiliani

**Affiliations:** ^1^Ball State University, Muncie, IN, USA; ^2^Jackson State University, Jackson, MS 39213, USA; ^3^Ball State University, Muncie, IN 47306, USA; ^4^Virginia Commonwealth University, Richmond, VA 23284, USA

**Keywords:** Tobacco, Smoking, Adolescent, School health, Prevention, Risk behavior

## Abstract

**Background:** Adolescent tobacco use in India has increased substantially within the past few decades. Schools can serve as an important avenue for prevention education, but little is known about the current practices of Indian schools in relation to tobacco use prevention education.

**Methods:** To assess the extent and influence tobacco use prevention education in Indian schools,we analyzed the Global Youth Tobacco Survey data for India, which was a population-based study of a national random sample of 10112 students from 180 private and public schools.Variables such as student demographic profile, tobacco use behavior, perceptions about tobacco use, and exposure to school-based tobacco use prevention education were considered for analyses.

**Results:** Prevalence of any form of tobacco use (14%) and current smoking (8%) was found to differ by gender. A quarter of the students believed that boys who smoke are more attractive or have more friends compared to non-smokers, and almost half of the students reported that smoking and health were never discussed as a part of a lesson in school. The association between school-based prevention education and tobacco use behavior was assessed (after adjustment forage, gender, and parental smoking). Students who were educated in school about tobacco use and its effects were significantly more likely to have negative attitude toward tobacco use and less likely to report use of tobacco.

**Conclusion:** School-based tobacco use prevention education has beneficial influence on adolescents in India. Given the early age of initiation of tobacco use, school curricula in India should emphasize on tobacco use prevention education.

## Introduction

Worldwide, tobacco use remains a leading cause of mortality and morbidity. Particularly, tobacco use in youth has become a significant global health problem. Nearly 20% of 13–15 year olds worldwide use some type of tobacco products.^1,2^ Among adolescents who smoke cigarettes, more than 25% smoked their first cigarette before the age of 15 years.^2^ Smoking related disease risk increases with early onset tobacco use and smoking during adolescence causes profound health problems, both during adolescence and in adulthood due to continued smoking. Tobacco use has been consistently linked with heart disease, cancers, and premature mortality.^2-7^

Frequently, smoking and tobacco use initiation occur during adolescence and continue into adulthood. Given the early age of initiation of tobacco use and smoking, prevention initiatives should target youth with particular emphasis on school-based prevention education.^3-7^ Such initiatives and educational interventions are especially in need for low and middle income countries because of the growing prevalence of tobacco use by youth in these countries. In addition, the current generation of children in low and middle-income countries will be the largest population in history to make transition to adolescence and adulthood.^1,2,8,9^ For example, almost a third of India’s population is in the age group of 1-19 years comprising one of the world’s largest population of children for any country.^8,9^ Also, according to various estimates, 5%-25% of Indian adolescents currently use or have ever used tobacco in various forms (e.g. chewing and smoking.^3-6^However, little is known about tobacco use prevention education practices in Indian schools and the influence of such education. Thus, the purpose of this study was to estimate the nature and extent of school-based tobacco use prevention education, to examine the attitudes about smoking and smoking behaviors, and to assess the influence of school based tobacco use prevention education on smoking related attitudes and behaviors in a large national random sample of adolescents in India.

## Material and Methods

### Participants and procedures

Data from the Global Youth Tobacco Survey (GYTS 2009) of India was used for this study. The GYTS was a school-based survey of a national random sample of students in 180 private and public schools in India. A 2-stage cluster sample design was used to produce representative data for India. A total of 10 112 students aged 13-15 completed the India GYTS survey with an overall response rate of 79.6%.^1,2,10^ Data files for this study were provided by the Centers for Disease Control and Prevention (CDC) and are publicly available without identifying information about the study participants. Each participating country for the global youth tobacco survey followed parental permission and relevant ethical procedures. We used the publicly available India data files for our analyses and therefore, no additional institutional clearance was required.

### Measures

The survey had questions about students’ attitudes and knowledge towards tobacco use, access to and use of tobacco products, exposure to tobacco smoke, and school curriculum on tobacco use prevention. To assess current smoking status or any form of tobacco use, participants were asked “During the past 30 days, on how many days did you smoke (e.g. cigarettes or bidis) or used any tobacco product (e.g. chewing or smoking)?” In this study, current smokers or tobacco users were those who reported having smoked or used any tobacco on one or more days during the last 30 days preceding the survey. To assess the education provided to students on tobacco use prevention, we used the following survey questions in our analysis: “During this school year, were you taught in any of your classes about the dangers of smoking?”, “During this school year, did you discuss in any of your classes the reasons why people your age smoke?”, and “During this school year, were you taught in any of your classes about the effects of smoking, like it makes your teeth yellow, causes wrinkles, or makes you smell bad?”. The response options for these questions were ‘yes’, ‘no’, and ‘not sure’. Another survey question was used in our analysis to assess the frequency of tobacco use prevention education in schools- “How long ago did you last discuss smoking and health as part of a lesson?” The response options were ‘never’, ‘this term’, ‘previous terms’.

Majority of the countries involved in the global survey of tobacco use by youth used the measures included in the India survey that we examined for this study. The measures have been validated and assessed for reliability by the World Health Organization (WHO) and the US CDC. More details about methods have been previously published.^1,2,10^

### Data analysis

Data from the study were analyzed using SPSS 23.0 (IBM Inc., Armonk, NY, USA), using the complex sample survey analysis procedures. Data analysis included descriptive statistics with a report of the appropriate frequencies to describe the responses to the questionnaire items related to tobacco use and school-based education on tobacco use prevention. Logistic regression analyses were conducted to assess the relationship between school-based prevention education (independent predictor variable) and tobacco use behavior, knowledge, and perceptions (outcome variable). All the analyses were adjusted for age and gender of study participants and parental smoking as there is evidence available for influence of parental smoking on youth tobacco use behavior and attitudes.^1,2,11^ Statistical significance was set *a priori* at *P* < 0.05

## Results

The majority of the study participants were females (51%) and students from grades 8, 9, and 10 were almost equally represented in the final sample (range 31%-35%) (Table 1). More than 1 in 10 (14%) students reported any form of tobacco use (e.g. chewing or smoking) with significant differences between males and females (19% vs. 8%, *P* < 0.01) and 8% of students reported current smoking (i.e. cigarettes or bidi) with males significantly more likely to smoke than females (11% vs. 4%, *P* < 0.01). With regards to the intent of smoking in future, majority of the students (85%) affirmed that they will definitely not smoke next year, within the next 5 years, or even if their best friend was to offer cigarettes (Table 1).

Students’ perceptions about smokers were assessed and almost a quarter of students believed that boys who smoke are more attractive (23%) or have more friends (25%). Also, more than a tenth of the students believed that girls who smoke are more attractive (16%) or have more friends (14%). The majority of students (72%) believed that smoking makes a person lose weight and 8% believed that smoking could cause weight gain. Students responded to knowledge items and the majority agreed that smoking cigarettes (83%), secondhand smoke (80%), and chewing tobacco (80%) are harmful. However, less than half (43%) believed that smoking is difficult to quit. Students were asked about the timing of tobacco use prevention education in schools and almost half (48%) reported that smoking and health were never discussed as a part of a lesson in school (Table 1).

In logistic regression analyses, first, we compared students who never received any education with those who received education in this term or previous terms about smoking and health (Table 2, column 3). Students who received education in this term were significantly less likely to believe that boys who smoke are more attractive (AOR = 0.62, 95% CI = 0.51-0.77, *P* < 0.01) or girls who smoke are more attractive (AOR = 0.59, 95% CI=0.48-0.73, *P* < 0.01). Compared to those who never received education, those who received it in this term or in previous terms were significantly more likely to believe that smoking or chewing tobacco is harmful, smoke from other people’s cigarettes is harmful, and smoking is hard to quit. Similarly, those who received education in this term or previous terms were less likely to be current smokers and had greater probability of denying smoking intentions in the next year, next 5 years, or if a best friend was to offer cigarettes. The associations were stronger for students who reported receiving education in this term of school. Associations were also significant for those students who received education in earlier terms, but weaker compared to those who received in this term.

Students who did not receive education this year were compared with those who received education this year on specific topics (e.g. effects of smoking, reasons why people smoke, and dangers of smoking) (Table 2, column 4). Students who discussed in class this year the effects of smoking and dangers of smoking were significantly less likely to believe that boys and girls who smoke have more friends, less likely to believe that girls who smoke are attractive, more likely to believe that chewing tobacco is harmful, and more likely to agree that smoking is difficult to quit once started. Students who discussed in class this year the reasons why people smoke, the effects of smoking, and dangers of smoking were significantly: more likely to believe that smoking cigarettes is harmful and second hand smoke is harmful, more likely to believe that smoking causes weight loss, more likely to deny any intentions to smoke within the next year, next 5 years, or even if a best friend was to offer cigarettes. Compared to those who did not receive education, current smoking behavior was statistically significantly lower in those who received education: in this academic term (AOR = 0.12, 95% CI = 0.08-0.17, *P* < 0.01), in previous academic terms (AOR = 0.36, 95% CI = 0.24-0.54, *P* < 0.01), and on topics such as the reasons people smoke (AOR = 0.60, 95% CI = 0.46-0.79, *P* < 0.01).

## Discussion

This study confirms the prevalence of any form of tobacco use (14.3%) in Indian adolescents that is well within the prevalence range estimated by previously published studies from India.^1-6^ Also, this study assessed the prevalence of school-based tobacco use prevention education. A large proportion of students have never discussed smoking and health in school, the reasons why people of their age smoke, and the effects of smoking. This is disconcerting because previous studies within and out of India have shown the positive and beneficial effects of school-based prevention education.^6,12-15^ Another pertinent finding of the study is the robust association between exposures to school-based education and positive attitudes and healthy behaviors with regards to tobacco use. Both frequency and content of education can influence adolescents’ attitudes and behaviors on tobacco use to the extent that school-based education was a better predictor of tobacco use behavior and attitudes attenuating the influence of parental smoking.

Schools across India should implement a standardized curriculum on smoking prevention education. Such education should be periodic, sustained, and culturally relevant to reap long-term benefits of reduction in youth tobacco use. In addition, parents and school administrators should also be included in the process of educating youth to prevent health risk behaviors.^3-8,12-15^ Tobacco use prevention education should be incorporated in science, biology, and health curriculum in Indian schools starting early in elementary or middle schools. Also, there should be a discussion about why students smoke, the dangers of smoking, and the fact that tobacco is one of the most highly used addicting substances with severe impact on health. Myths and misperceptions such as smokers have more friends or they are more attractive should also be addressed.^3-6,13-15^ The Government of India should consider evidence based practices to effectively deal with the youth tobacco epidemic using schools as critical avenues for prevention and education. Historically, the health agenda for youth in India always had and still has a weak emphasis on tobacco use prevention (especially with regards to school-based prevention education). For example, programs such as the School Health Program and Adolescence Education Program have mostly emphasized on problems such as poor nutrition, infectious disease, immunization, reproductive health, and screening for disease.^7,8,16,17^ Recently, some advancements have been made by vigorous efforts from NGOs and government agencies to advocate for school-based tobacco use prevention education in India.^18,19^

Tobacco use in adolescents is a complex problem that requires multifaceted interventions including parental supervision and awareness about adolescent behaviors, school-based prevention education, and community legislation to make it difficult for youth to access tobacco products.^3-6,13-15,18-21^ Fortunately, there have also been major advancements in tobacco prevention education from first generation knowledge-based interventions to second generation skill-based interventions (such as refusal skills, problem-solving skills etc) to now third generation, theory-based interventions. Such theory based interventions must be implemented in schools and school personnel in India will have to be the prime agents of change to implement evidence based curricula and prevention practices for adolescents.^14,15,18,20,21^ Preventing and reducing adolescent tobacco use in India would also require the involvement of parents, pediatricians, school personnel, and attention from policymakers. Currently, given the results of our study, it can be said that much remains to be done to prevent tobacco use in youth of India.

The results of this study suffer from all the traditional limitations of a cross-sectional research design (e.g. reliance on self-reported behaviors, recall bias in participants and socially desirable responses, and the inability to establish cause and effect relationships). Despite the limitations, the strength of this study lies in the large national sample of participants, a feature of the study that can enable external validity and generalizability of findings to the student population across India.

## Conclusion

This study was an attempt to assess attitudes about tobacco use, tobacco use behavior, and the influence of school-based prevention education in Indian adolescents. The results are important both to assess adolescents’ views on smoking and to establish control measures for prevention of tobacco use in adolescents. We found an association between the content and frequency of tobacco use prevention education (in schools) and attitudes about smoking and tobacco use behavior. Unfortunately, the majority of the adolescents in Indian schools are not being regularly educated on tobacco use prevention. Increasing the outreach and frequency of education and improving the content of anti-tobacco education in schools for adolescents holds great potential to prevent students from smoking in India.

## Ethical approval

We used the publicly available India data files for our analyses and therefore, no additional institutional clearance was required.

## Competing interests

 We declare no conflicts of interest.

## Authors’ contributions

JK, MS, DH, and JT contributed to the conception and design of the study and the writing and critical editing of the manuscript. JK and MS analyzed and interpreted the data. DH and JT provided critical revision of the article JK, MS, DH, and JT have approved this submission.

## Funding

The funding for the multi-country Global Youth Tobacco Survey was provided by the WHO-tobacco free initiative and National Cancer Institute, USA. We are also grateful to the US CDC for providing the data files. The funding agencies did not contribute to the writing of this manuscript or data analysis.

## Acknowledgements

Dr. Khubchandani is a recipient of Center for International Development Fellowship and Diversity Associates Scholarship from Ball State University.

**Table 1:** Students’ characteristics, tobacco use behavior and perceptions, and exposure to school-based tobacco use prevention education

**Demographics**	**No. (%)** ^a^
Gender	
Male	4822 (48)
Female	5214 (52)
Age	
13 years old	3220 (32)
14 years old	3755 (37)
15 years old	3137 (31)
Grade	
8th grade	3106 (31)
9th grade	3563 (35)
10th grade	3390 (34)
**Tobacco Use Behavior and Intention**	
***Adolescent Study Participants: Current Tobacco Use***	
Any tobacco Use	1295 (14)
Smoking (e.g. cigarette or bidi)	707 (8)
Other tobacco use (e.g. chewing)	845 (9)
***Adolescent Study Participants: Tobacco Use Intention***	
At any time during the next 12 months, do you think you will smoke a cigarette	
Definitely not	8653 (85)
Probably not/ probably yes	1250 (12)
Definitely yes	135 (2)
Do you think you will be smoking cigarettes 5 years from now	
Definitely not	8669 (85)
Probably not/ probably yes	1289 (13)
Definitely yes	135 (2)
If one of your best friends offered you a cigarette, would you smoke it	
Definitely not	8574 (85)
Probably not/ probably yes	1161 (12)
Definitely yes	262 (3)
Parental smoking	
Both parents smoke	506 (5)
Only father smokes	1971 (19)
Only mother smokes	199 (2)
Students’ perceptions	
*Boys* who smoke cigarettes have more *Friends*	2486 (25)
*Girls* who smoke cigarettes have more *Friends*	1380 (14)
Smoking cigarettes makes *Boys* look more *Attractive*	2275 (23)
Smoking cigarettes makes *Girls* look more *Attractive*	1621 (16)
Smoking makes you lose weight	7278 (72)
Smoking makes you gain weight	746 (8)
Students’ knowledge	
Smoking cigarettes is harmful	8433 (83)
Smoke from other people’s cigarettes is harmful	8076 (80)
Once some has started smoking, it would be difficult to quit	4392 (43)
Chewing tobacco is harmful	8111(80)
**Exposure to School Based Prevention Education**	
Frequency of education	
Never discussed smoking and health as part of a lesson	4805 (48)
Discussed smoking and health as part of a lesson (*this term*)	2392 (24)
Discussed smoking and health as part of a lesson (*previous terms*)	2882 (28)
Content of education	
Discussed during this year in class why people of your age smoke	3834 (38)
Discussed during this year in class the effects of smoking (e.g. yellow teeth and bad smell)	5623 (56)
Taught during this year in class about the dangers of smoking	6457 (64)

^a^ Percent values rounded to the closest digit and numbers may not add up to 100% due to missing values. N[total participants] = 10 112.

**Table 2:** School based prevention education: association with tobacco use attitudes, knowledge, and behavior

**Student Perceptions, knowledge, and behavior**	**Never discussed (Ref. group)**	**Periodicity/frequency of education**		**Content of education (discussed in class this year)**		
		**Discussed smoking & health as a Lesson (this term) AOR (95% CI)**	**Discussed smoking & health as a lesson (previous terms) AOR (95% CI)**	**Discussed the effects of smoking AOR (95% CI)**	**Discussed why people of their age smoke AOR (95% CI)**	**Discussed dangers of smoking AOR (95% CI)**
**Perceptions**						
Smoker Boys Have More Friends	1(Ref)	0.95(0.71-1.26)	1.28(0.94-1.76)	0.57(0.48-0.70)^**^	0.70(0.56-0.89)^**^	0.55(0.45-0.72)^**^
Smoker Girls Have More Friends	1(Ref)	0.96(0.72-1.28)	1.19(0.94-1.50)	0.81(0.68-0.96)^*^	1.02(0.83-1.27)	0.54(0.44-0.69)^**^
Smoker Boys are More Attractive	1(Ref)	0.62(0.51-0.77)^**^	0.89(0.68-1.15)	0.81(0.61-1.08)	0.88(0.71-1.10)	0.63(0.53-0.77)^**^
Smoker Girls are More Attractive	1(Ref)	0.59(0.48-0.73)^**^	1.42(0.98-2.01)	0.75(0.58-0.96)^*^	0.89(0.68-1.17)	0.63(0.47-0.85)^**^
Smoking makes you Lose Weight	1(Ref)	1.07(0.81-1.44)	1.10(0.79-1.52)	2.27(1.76-2.81)^**^	1.45(1.12-1.89)^*^	1.82(1.46-2.26)^**^
Smoking makes you Gain Weight	1(Ref)	0.59(0.41-0.84)^*^	0.81(0.51-1.28)	1.76(1.24-2.49)^**^	1.37(1.02-1.84)^*^	1.29(0.99-1.68)
**Knowledge**						
Smoking Cigarettes is Harmful	1(Ref)	2.01(1.59-2.54)^**^	1.92(1.44-2.56)^**^	1.69(1.27-2.24)^**^	1.27(1.06-1.53)^*^	2.30(1.90-2.79)^**^
Smoke from other peoples cigarettes is harmful	1(Ref)	1.91(1.46-2.51)^**^	1.33(1.01-1.74)^*^	1.88(1.60-2.18)^**^	1.21(1.01-1.45)^*^	2.60(2.22-3.07)^**^
Once some has started smoking, it would be difficult to quit	1(Ref)	2.46(1.89-3.21)^**^	1.36(1.08-1.71)^*^	1.32(1.02-1.70)^*^	1.04(0.82-1.31)	1.43(1.12-1.83)^*^
Chewing Tobacco is Harmful	1(Ref)	2.21(1.70-2.86)^**^	1.98(1.64-2.40)^**^	1.65(1.36-1.98)^**^	1.07(0.90-1.28)	2.13(1.80-2.53)^**^
**Tobacco Use Behavior & Intention**						
Current Smoking	1(Ref)	0.12(0.08-0.17)^**^	0.36(0.24-0.54)^**^	0.88(0.63-1.23)	0.60(0.46-0.79)^**^	0.82(0.49-1.40)
Would not smoke even if one of the best friends offered a cigarette	1(Ref)	7.23(4.85-10.80)^**^	3.03(1.99-4.63)^**^	3.42(2.21-5.30)^**^	1.78(1.29-2.45)^**^	1.95(1.12-3.39)^*^
Will not smoke in the next 12 months	1(Ref)	7.10(4.21-11.98)^**^	1.80(1.07-3.06)^*^	3.61(2.34-5.54)^**^	1.77(1.21-2.58)^**^	2.83(1.70-4.72)^**^
Will not smoke in the next 5 years	1(Ref)	7.04(4.77-10.37)^**^	2.06(1.27-3.32)^**^	3.36(2.07-5.55)^**^	1.70(1.10-2.62)^*^	3.06(1.63-5.75)^**^

AOR = Adjusted odds ratios (95% confidence interval) indicates the probability of having certain perceptions, knowledge, or behaviors. The rows indicate outcome variable and columns indicate predictor variables (i.e. education is predictor and perceptions, knowledge, and behavior are outcomes). AOR = Adjustments were made for students’ gender, grade,age, and parental smoking. * *P* < 0.05 and ** *P* < 0.01. AOR >1 indicates higher probability of an outcome and AOR<1 indicates lower probability of an outcome (e.g. knowledge, behavior, or perception).
